# Morphology of the Human Pineal Gland Studied by Freeze-Fracturing in Scanning Electron Microscopy

**DOI:** 10.3390/life14121617

**Published:** 2024-12-06

**Authors:** Simona Polakovičová, Ján Líška, Ivan Varga, Paulína Gálfiová

**Affiliations:** Institute of Histology and Embryology, Faculty of Medicine, Comenius University in Bratislava, Špitálska Street 24, 813 72 Bratislava, Slovakia

**Keywords:** pineal gland, acervulus, pinealocyte, ultrastructure, scanning electron microscopy

## Abstract

The human pineal gland is the largest producer of the hormone melatonin. Pineal acervuli (brain sand), calcified concretions in the pineal gland, have long been studied because of their association with ageing, melatonin production, and neurological disorders. The solid inorganic matter of the hydroxyapatite crystals often renders sample sectioning impossible, to the extent that the sections lose value. Technically, freeze-fracturing has revealed the detailed structure and cell relationships without tissue damage. In our electron microscopic study, samples of the human pineal gland were obtained during autopsy from 20 donors with mean age 69 years. Samples underwent freeze-fracturing and standard histological procedures, and were analysed by scanning electron microscopy (SEM) in high vacuum. Based on our results, freeze-fracturing enabled identification of a mulberry-like acervulus topography. The acervuli were situated in specific “nest-like” structures, which were surrounded by pinealocytes, interstitial cells, and nerve fibres. A fractured surface of the intrapineal acervuli exhibited a regular lamellar structure. Freeze-fracturing the pineal gland and imaging by SEM enabled complex structural analysis. This approach permits viewing the surface acervuli spherical and internal lamellar architecture. Our results confirmed that the parenchyma of this small but important gland contains two types of acervuli, depending on their size: non-aggregated and aggregated. We propose to include these forms of acervuli in the new edition of the *Terminologica Histologica*. In conclusion, pineal gland freeze-fracturing by SEM is suitable for complex structural analysis. Our description of our methods can be a guide for other scientists who want to study the pineal gland with electron microscopy methods.

## 1. Introduction

The pineal gland, both in humans and animals, was an enigmatic organ for centuries. René Descartes (1596–1650), the famous French philosopher, considered the human pineal gland to be the control centre of the body and the seat of the soul [1]. In the following centuries, the human pineal gland was classified as a rudimentary vestigial organ, the remaining of the lower vertebrate’s third eye. The consideration of the pineal gland as having no physiological function in mammals lasted until the late 1950s. The neuroendocrine nature of the pineal gland was finally proven after the isolation of the melatonin hormone in 1958 and a subsequent discovery that melatonin acts as a potent neurotransmitter in the central nervous system, turning the pineal gland into a “biological clock” [2].

The human pineal gland is a neuroendocrine tissue (7 mm × 6 mm × 3 mm) located on the roof of the third ventricle, connected to the diencephalon via the pineal stalk. In mammals, including humans, this gland is rich in blood vessels and secretes the hormone melatonin [3,4]. The pineal gland is directly connected to the central nervous system. The principal cells of this gland—the pinealocytes—secrete melatonin, which regulates the circadian rhythm. Two distinct types of pinealocytes can be identified at the ultrastructural level: type I (light) cells and type II (dark) cells. In addition to the two previously described types, Hussain [5] identified a third type, characterized by an elongated body and a longest diameter ranging from 12–32 µm (the diameter of types I and II is between 7 and 11 µm). Interstitial glial cells (classified as astrocytes) are located between the pinealocytes and constitute ca. 5% of the cells in the gland [6]. The pineal gland is one of the circumventricular organs that lacks a blood–brain barrier.

The Italian anatomist Giovanni Battista Morgagni (1682–1771) was the first to use the term “brain sand” (referring to acervuli). Morgagni described a relationship between the existence of acervuli in the pineal organ and the existence of mental deficiency or mental retardation [2]. Nowdays, acervuli are considered a normal physiologic phenomenon—calcified concretions—in the pineal gland. They are detectable not only microscopically, but also by skull radiography and computer tomography (CT) scans. Pineal calcification is seen on skull radiography in 61% and on CT scans in 83% of patients over the age of 30 years [7]. This phenomenon is more frequently observed in women prior to menopause than in men [8,9]. At the microscopic level, Antic et al. [8] identified two distinct types of human pineal acervuli: extrapineal and intrapineal. The extrapineal acervulus is situated within the connective tissue capsule and exhibits a round or oval shape, as well as a lamellar structure. The intrapineal acervulus is characterised by a homogeneous structure and a spherical shape. Duvernoy et al. [10] observed larger and irregular acervuli and mulberry-like structures in older individuals. Kim et al. [11] observed the growth patterns of acervuli in the human pineal gland. He discovered that the majority of acervuli measuring less than 200 µm were non-aggregated, whereas those exceeding 200 µm were aggregated, with the size varying in accordance with age. The acervuli of the rat pineal gland are typically composed of S, Na, Mg, Sr, Ca, and P and hydroxyapatite crystals [11,12,13]. The composition of this structure remains a subject of ongoing research. The modern method of energy-dispersive X-ray analysis enables study of the acervuli composition. Such work is pertinent because the degree of calcification has been linked to a number of neurodegenerative disorders, including multiple sclerosis [14], epilepsy [15], and Alzheimer’s disease [16].

The precise mechanism of acervulus formation remains uncertain, although several hypotheses have been proposed [17]. Lukaszyk and Reiter [18] propose that pinealocytes secrete polypeptides into connective tissue, which are transported by carrier protein neuroepiphysin and replace calcium. Krstic [13] indicates that acervuli are derived from pinealocytes. The cytoplasmic matrix, mitochondria, and endoplasmic reticulum undergo transformation into acervuli after adding hydroxyapatite crystals. The cells undergo degeneration and extrude this material into the extracellular space. The primary component of the acervulus structure is hydroxyapatite, which is also the main component of bone tissue [17,19]. Another indicator that an acervulus resembles an osteon is the layering of inorganic substances in a pattern of concentric lamellae. Tan et al. [17] hypothesized that acervulus formation is a physiological process, distinct from pathological bone formation. An alternative hypothesis posits that formation of the pineal acervuli is associated with pathological disorders and ageing. Maslinska et al. [20] demonstrated that calcification initiates exclusively in degenerating regions of the pineal gland in newborns and young children. The process by which calcification of the pineal gland occurs remains unknown. However, some studies considered the involvement of mast cells [20]. In pathologically altered tissue, activated mast cells mobilise calcium from intracellular stores [20,21]. The majority of these cells are situated in close proximity to blood vessels, a location that enables release of mast cell products (histamine and nitric oxide) that regulate microcirculation and vascular permeability. Calcified mast cells form morula-like structures [20].

Given the technical difficulties involved in removing the pineal gland from a living human subject, study of its structure is largely confined to post-mortem examination. Human pineal glands are usually harvested through autopsy. A number of scientists have used tissue derived from rats, donkeys, and monkeys [22,23]. The aim of our study is a detailed scanning electron microscopy (SEM) study of the human pineal gland with a special focus on the ultrastructure of the acervuli.

## 2. Materials and Methods

Samples of the human pineal gland were obtained from cadavers of 20 donors, comprising 10 women and 10 men, at the Institute of Pathological Anatomy of the Faculty of Medicine, Comenius University in Bratislava, Slovakia. The mean age of all donors, irrespective of gender, was 69 years. The mean age of the female participants was 71 years ± 7.6. The youngest woman was 60 years of age, while the oldest was 82 years of age. The mean age of the male participants was 67 years ± 13.5. The youngest man was 50 years of age, while the oldest was 93 years of age. In order to observe biological samples, it is necessary to prepare them for high vacuum conditions. One of the ways that specimens can be prepared for observation in the SEM is by chemically treating them. The combination of physical freeze-fracturing of the sample to expose artificial surfaces and subsequent chemical treatment is a modification of the basic chemical method. Chemical processing of biological samples involves successive steps such as excision, two-step chemical fixation, rinsing in phosphate buffer solution, dehydration, critical point drying, and sputtering by conductive elements. When studying parenchymal organs such as the pineal gland, we artificially exposed the internal structure by freeze-fracturing in a mixture of dry ice and acetone (temperature approximately −79 °C). We did not rinse the samples before breaking them, as this would cause artefacts. The samples from autopsy were put on the dry ice (solid carbon dioxide) surface immediately, resulting in physical fixation. This step provided a preventive treatment of the samples against autolysis. After fracturing the sample, prior to chemical fixation an appropriate amount of the total volume was handled carefully to protect the fracture surface. The surfaces revealed by sample fracturing have been very useful in studying the detailed structure of the gland, both the inorganic and the organic components. After fracturing, the specimen was immediately fixed by immersion in a chemical fixative consisting of 3% glutaraldehyde solution in phosphate buffer (0.2 molarity, pH 7.3) at room temperature for 4 h. After rinsing in a buffer solution, a further fixation (postfixation) was carried out in a 1% solution of osmic acid in Millonig buffer (0.1 molarity, pH 7.3). Glutaraldehyde preserves the structure of proteins and osmium salts, fixing the lipid components of the cytoplasmic membranes, which is essential for the stability of the cell surface shape. After fixing and rinsing off the fixative solution residues, we carefully dehydrated the sample using a graded ethanol series up to absolute ethyl alcohol. The displacement of alcohol and drying of the sample without changing the surface tension was ensured by the critical point drying method (CPD). For this purpose, we used a Leica CPD 300 machine. After the sample was dried, we mounted the sample to a conductive stub by adhesive carbon tapes and deposited a conductive layer with a thickness of 15–20 nm, depending on the surface roughness (in our case, a gold–palladium mixture) using the Leica EM ACE 200. Samples prepared in this way were examined with a ZEISS EVO LS 15 scanning electron microscope at the Institute of Histology and Embryology, Medical Faculty, Commenius University in Bratislava. The study protocol was approved by the relevant ethical committee, namely the Ethics Committee of the Faculty of Medicine, Comenius University and University Hospital in Bratislava, Old Town Hospital (project number EK62/2019).

## 3. Results

**Figure 1:**
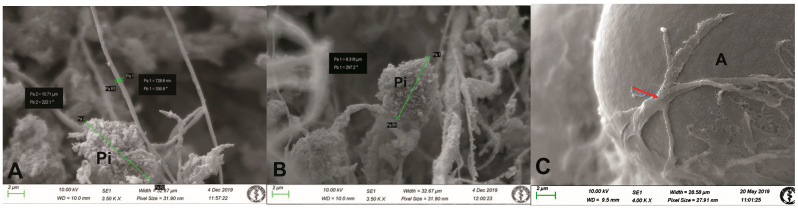
Scanning electron microscopy images of the human pineal gland using a modified freeze-fractured sample procedure. (**A**,**B**) Pinealocytes (Pi) with round or elongated shape [measuring from 8.3 µm (**B**) to 10.71 µm (**A**)], surrounded by thin non-myelinated nerve fibres, measuring 0.7 µm in diameter; orig. magn. 3500×. (**C**) Intimate contact of an interstitial glial cell (astrocyte) (arrow) with inorganic material of an acervulus (A), orig. magn. 4000×.

We investigated the architecture of the human pineal gland at the electron microscopic level. SEM enabled us to clearly distinguish the pinealocytes and interstitial glial cells (astrocytes) found in the parenchyma. Pinealocytes exhibited as larger cells of round or irregular shape, with long processes. The mean diameter of the pinealocytes was 6–11 µm (Figure 1A,B). We did not identify pinealocytes exceeding 12 µm. The interstitial glial cells (astrocytes) were smaller than the pinealocytes and exhibited an elongated body and long processes (Figure 1C).

**Figure 2:**
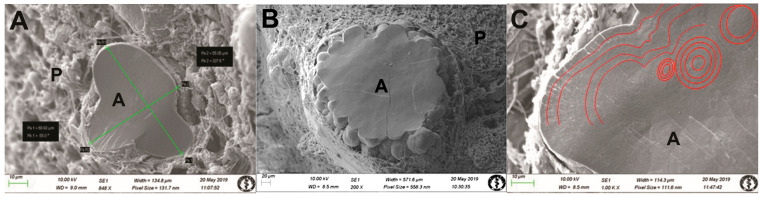

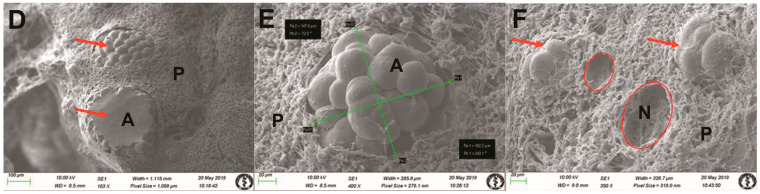
Scanning electron microscopy images of the human pineal gland using a modified freeze-fractured sample procedure. (**A**) The structure was comprised of artificially fractured, non-aggregated, irregular mulberry-like acervuli arranged in a “nest-like” configuration surrounded by pinealocytes, interstitial glial cells (astrocytes), and nerve fibres. The dimensions are 55 µm × 60 µm. A—acervulus, P—parenchyma, orig. magn. 848×. (**B**) The structure was characterised by the presence of an artificially fractured mulberry-like acervulus, situated within a “nest-like” configuration and displaying a regular pattern. This was surrounded by a complex network of cells including pinealocytes, interstitial glial cells (astrocytes), and nerve fibres. A—acervulus, P—parenchyma, orig. magn. 200×. (**C**) The detailed structure of the aggregated mulberry-like acervuli on the artificial fractured area. The structure appeared to exhibit growth patterns or concentric rings, as observed under a magnification of 1000×. (**D**) The parenchyma of the gland contained both intact and artificially cut mulberry-like regular acervuli (red arrows). A—acervulus, P—parenchyma, orig. magn. 103×. (**E**) The non-aggregated mulberry-like acervuli were situated within a “nest-like” structure surrounded by pinealocytes, interstitial glial cells (astrocytes), and nerve fibres. The dimensions of the structure are 148 µm × 162 µm. A—acervulus, P—parenchyma, orig. magn. 400×. (**F**) Following the removal of the acervuli (red arrows), the location appeared to resemble “nest-like” structures (circles) created as an imprint of the parenchyma surrounded by pinealocytes, interstitial glial cells (astrocytes), and nerve fibres. N—nest, P—parenchyma, orig. magn. 350×.

**Figure 3:**
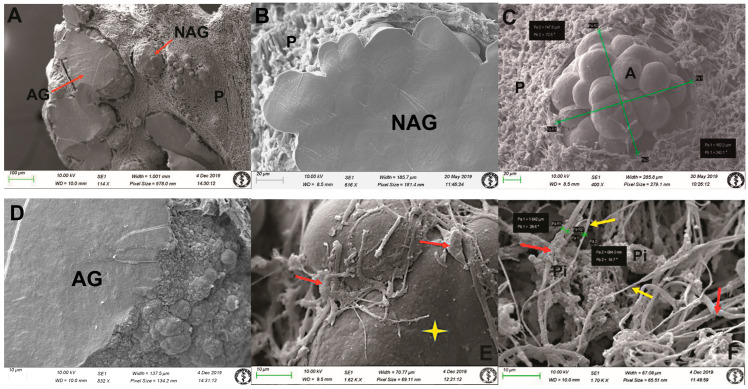
Scanning electron microscopy images of the human pineal gland, obtained through a modified freeze-fractured sample procedure. (**A**) Overview image of artificial surface with the presence of aggregated (AG) and non-aggregated (NAG) acervuli embedded in the parenchyma (P) of the gland, orig. magn. 114×. (**B**) Representative surface of an artificially fractured non-aggregated (NAG) acervulus within the parenchyma (P), orig. magn. 616×. (**C**) Surface of non-aggregated (NAG) mulberry-like acervuli (A) (size 148 µm × 162 µm) embedded in parenchyma (P), orig. magn. 400×. (**D**) Representative surface of both artificially fractured and natural surface of aggregated (AG) acervuli, orig. magn. 832×. (**E**) The arrows indicate intimate connection of pinealocytes with nerve fibres to the acervulus (yellow star), orig. magn. 1620×. (**F**) A tangle of non-myelinated nerve fibres (yellow arrows) within the parenchyma of the gland, in close proximity to pinealocytes (Pi). The thickness of the thinner (yellow arrows) to thicker (red arrows) nerve fibres varied from 0.7–1.6 µm, orig. magn. 1700×.

At the electron microscopic level, the majority of intrapineal acervuli were homogeneous, with varying sizes observed within the parenchyma of the gland (Figure 2 and Figure 3). Pinealocytes and interstitial glial cells (astrocytes) exhibit long processes that communicate with inorganic acervuli (Figure 1C and Figure 3E).

We used freeze-fracturing in conjunction with SEM to obtain a detailed morphological classification. Regarding three-dimensional structure, this technique permitted identification of mulberry-like acervuli (Figure 2B,D–F and Figure 3A–C). The intrapineal acervuli exhibited a regular lamellar structure in a circular orientation, corroborated by a higher magnification analysis (Figure 2C and Figure 3B). The acervuli were situated in specific “nest-like” structures, which were surrounded by pinealocytes, interstitial cells, and nerve fibres (Figure 2D–F and Figure 3C). In our samples, the size of the acervuli exhibited a considerable degree of variability, with some measuring as little as 10 µm and others reaching a maximum diameter of 600 µm. Additionally, our study revealed the presence of numerous thin nerve fibres within the parenchyma between cells and acervuli (Figure 3F). The fibres observed in our samples between the pinealocytes and interstitial glial cells (astrocytes) were predominantly thin, with an average diameter of approximately 0.7 µm. The thickest fibres measured 1.6 µm in size (Figure 1A,B and Figure 3F). We propose that these fibres correspond to the autonomic nervous system due to their morphology.

## 4. Discussion

One of the techniques used in SEM is freeze-fracturing, whereby the surface of the examined sample can be gently revealed by breaking it using a mixture of dry ice and acetone before fixation. This method enables detailed study of structural intracellular relationships without damage caused by tissue sectioning, simultaneously with preservation of hard inorganic components. One advantage of sample processing for SEM purposes is the possibility of further morphological analysis of artificial surfaces. Application of freeze-fracturing enabled the detailed structure of the parenchyma of the pineal gland to be revealed in a three-dimensional image. This technology enabled detailed study of not only the parenchyma itself, but also the acervuli. It is difficult to obtain a human pineal gland from a biopsy. More common is using animal tissue from a biopsy, which is less difficult to obtain and does not undergo autolysis. The small size and topography of the gland is another problem in obtaining human material.

In our research, we studied only human tissue—exclusively obtained by autopsy—that had undergone precise processing. We subsequently subjected the tissue to further processing by SEM. The structure of the acervuli at the quarry closely resembled the structure of osteons in human compact bone. As mentioned in the introduction, Antic et al. [8] described two types of acervuli with regard to their localization: within the gland (intrapineal) and outside the gland (extrapineal). We observed only intrapineal acervuli present in the gland parenchyma. The extrapineal acervuli were out of our focus. Intrapineal acervuli increase with age, with smaller subunits aggregating in a manner that forms larger subunits, resulting in the formation of mulberry-like structures. In accordance with size, the intrapineal acervuli come in two forms: non-aggregated and aggregated. Non-aggregated forms measure at least 150 µm and aggregated forms exceed 200 µm. A mixed acervulus is defined as having a dimension between 150 and 200 µm [8]. Our samples exhibited coexistence of both types of intrapineal acervuli within the intact parenchyma surrounding them. In the locations where we removed the material during processing, the imprints—which resembled nests—remained. Freeze-fracturing enabled us to understand the relationships between individual structures.

The precise mechanism of acervulus formation remains unclear. However, some researchers have proposed that formation may be changed during certain neurological and psychiatric disorders, such as chronic alcoholism, Alzheimer’s disease, and schizophrenia [22]. The hypothetic link between the pineal gland and some neurological diseases is based on fact that melatonin not only plays an important role in the regulation of circadian rhythms, but also acts as antioxidant and neuroprotector that may be of importance in ageing and Alzheimer’s disease [23]. Some studies have demonstrated that pinealocytes play a substantial role in acervulus formation (and melatonin production), whereas other studies have highlighted the importance of the stroma in acervulus formation. Nevertheless, it remains unclear how acervulus formation can be influenced, given that its increased amount is associated with premature ageing as a consequence of psychiatric disorders. Some animal studies in rats have transplanted the pineal gland to various body sites outside the third ventricle [17]. Ectopic gland transplantation appears to be beneficial to life from several perspectives. First, the normal in situ gland constantly produces high levels of melatonin, a hormone that protects the brain from oxidative stress. Second, secretion of melatonin in the cerebrospinal fluid of the third ventricle of the brain peaks at night and has a sharp decline compared with serum levels. The alteration might serve as an indicator of the biological rhythm of organisms. An ectopic transplanted pineal gland is devoid of these characteristics. Transplantation of the pineal gland to its physiological location on the roof of the third ventricle (in situ graft) represents an improved method for imitating its own activity. The pineal glands transplanted in this way survived due to revascularisation and partial reinnervation. However, the hormone melatonin, which is typically secreted at night, was produced at low levels and without a nighttime increase. Microdialysis permits measurement of melatonin levels. This method could facilitate decalcification of the pineal gland. Additionally, modified pinealocyte stem cells can be injected directly into the decalcified pineal gland [17].

The gland is innervated by sympathetic, parasympathetic, and central nerves. The primary mediators of the pineal gland’s hormonal activity are the sympathetic postganglionic nerve fibres, which lack a myelin sheath. These fibres are responsible for activating pinealocytes, which produce the hormone melatonin. Gregory et al. [24] revealed that these fibres correspond to the nervi conarii, which originate from the superior cervical ganglion. They are responsible for direct stimulation of pinealocyte secretion. These fibres form a plexus around the gland that produces norepinephrine. A considerable number of fibres in the gland parenchyma terminate freely between the pinealocytes, around vessels where they secrete norepinephrine to the pinealocytes overnight [25]. Based on the thickness of the nerve fibres, we also demonstrated the presence of a substantial number of unmyelinated nerve fibres. We observed these fibres in close connection with pinealocytes, supporting cells and the acervuli, as depicted in three-dimensional visualisation, which provides insight into the structure of this small but pivotal gland.

There are certain inconsistencies with regard to the existence and naming of certain structures in the pineal gland. In accordance with the current *Terminologica Histologica* [26], there are not two or even three types of pinealocytes, nor two different types of acervuli in accordance with their location (extrapineal and intrapineal), as mentioned by some researchers. The term “interstitial glial cells” is also incorrect; they are classified as astrocytes. As our research revealed two types based on the size of the acervuli (non-aggregated and aggregated), we propose that this classification be retained in the next edition of the internationally accepted histological nomenclature, the *Terminologica Histologica*.

The only limitation of our electron microscopic research is the group of older donors, without comparison with younger individuals. There is a problem to obtain the tissue from younger donors where we could expect a smaller number and different composition of acervuli. It is well-known from the results of other authors that the number and size of calcified concretions in the pineal gland increases with age [27,28,29,30]. However, in the scientific literature, there are also different research results. Surprisingly, Hasegawa et al. [31] found no calcification in some pineal glands of cadavers over 90 years old, which appeared indistinguishable from the pineal glands of younger individuals. This is also why the process of calcification within the pineal gland requires further research, especially regarding the influence of the rate of calcification on melatonin production.

## 5. Conclusions

The pineal gland is a central structure in the circadian system which produces melatonin under the control of the central biological clock. In the 21st century, it remains an enigmatic organ due to its atypical anatomical microstructure and complex functions. With improved technology, research conducted in the 20th and 21st centuries has led to a more comprehensive understanding of the morphology, development, and function of the pineal gland. The gland’s small size and specific location in an inaccessible area present challenges in obtaining samples. Our goal was to collect suitable material to avoid autolysis and preserve the tissue for SEM observation. Pineal gland freeze-fracturing by SEM is suitable for complex structural analysis. Our description of our methods can be a guide for other scientists who want to study the pineal gland with electron microscopy methods. SEM enables viewing both the surface spherical and internal lamellar architecture of acervuli. Due to the differing structural characteristics of the pineal gland between species, studying human gland morphology is primarily reliant on human material obtained from autopsy, which is more accessible than a challenging biopsy. Based on our results, we propose to incorporate two distinct types of acervuli within the next edition of the *Terminologica Histologica*.

## Data Availability

The original contributions presented in the study are included in the article, further inquiries can be directed to the corresponding author.
